# Performance of Qualitative and Quantitative Antigen Tests for SARS-CoV-2 Using Saliva

**DOI:** 10.3390/idr13030069

**Published:** 2021-08-24

**Authors:** Isao Yokota, Takayo Sakurazawa, Junichi Sugita, Sumio Iwasaki, Keiko Yasuda, Naoki Yamashita, Shinichi Fujisawa, Mutsumi Nishida, Satoshi Konno, Takanori Teshima

**Affiliations:** 1 Department of Biostatistics, Faculty of Medicine, Hokkaido University, Sapporo 060-8638, Japan; yokotai@pop.med.hokudai.ac.jp; 2 Division of Laboratory and Transfusion Medicine, Hokkaido University Hospital, N15, W7, Kita-ku, Sapporo 060-8638, Japan; takayo-72@huhp.hokudai.ac.jp (T.S.); sugitaj@med.hokudai.ac.jp (J.S.); sumio55@med.hokudai.ac.jp (S.I.); omiya@med.hokudai.ac.jp (K.Y.); n2639@med.hokudai.ac.jp (N.Y.); shinfuji@med.hokudai.ac.jp (S.F.); mutuni@med.hokudai.ac.jp (M.N.); 3 Department of Respiratory Medicine, Faculty of Medicine, Hokkaido University, Sapporo 060-8638, Japan; satkonno@pop.med.hokudai.ac.jp; 4 Department of Hematology, Faculty of Medicine, Hokkaido University, Sapporo 060-8638, Japan

**Keywords:** SARS-CoV-2, COVID-19, saliva, Lumipulse, Espline, ICA, CLEIA

## Abstract

The rapid detection of SARS-CoV-2 is critical for the prevention of disease outbreaks. Antigen tests such as immunochromatographic assay (ICA) and chemiluminescent enzyme immunoassay (CLEIA) can yield results more quickly than PCR. We evaluated the performance of ICA and CLEIA using 34 frozen PCR-positive (17 saliva samples and 17 nasopharyngeal swabs [NPS]) and 309 PCR-negative samples. ICA detected SARS-CoV-2 in only 14 (41%) samples, with positivity rates of 24% in saliva and 59% in NPS. Notably, ICA detected SARS-CoV-2 in 5 of 6 samples collected within 4 days after symptom onset. CLEIA detected SARS-CoV-2 in 31 (91%) samples, with a positivity of 82% in saliva and 100% in NPS. These results suggest that the use of ICA should be limited to an earlier time after symptom onset and CLEIA is more sensitive and can be used in situations where quick results are required.

## 1. Introduction

Rapid detection of SARS-CoV-2 is critical for the prevention and containment of COVID-19 outbreaks in communities. The “gold standard” of viral detection is quantitative reverse transcriptase polymerase chain reaction (PCR) using a nasopharyngeal swab (NPS). Self-collected saliva can be as effective as NPS, providing a major step for a type of screening that is much faster, and less inclusive and expensive [1,2,3,4]. Viral antigen detection is easy and can yield results quicker than PCR [5,6,7]. Herein, we evaluated the utility of immunochromatographic assay (ICA) and chemiluminescent enzyme immunoassay (CLEIA) in comparison with PCR.

## 2. Materials and Methods

We screened 343 samples that were the remainder of each sample after they had been used for PCR testing at our hospital and frozen at −80 °C. Among these (34 (17 NPS and 17 saliva) PCR-positive samples and 309 negative saliva samples), 78 samples had been tested in our previous study [8] and the remaining 265 samples were collected after that study. PCR-positive samples were taken as inpatients, while PCR-negative samples were taken as outpatients. Testing was performed at one site in our hospital. Frozen samples were thawed for this study and centrifuged at 2000× *g* for 5 min at 4 °C to remove debris. PCR tests were performed as described using StepOnePlus Real Time PCR System (Thermo Fisher Scientific, Waltham, MA, USA) [8], according to the manual by National Institute of Infectious Diseases (https://www.niid.go.jp/niid/images/epi/corona/2019-nCoVmanual20200217-en.pdf (accessed on 3 May 2020)).

ICA was performed only for PCR-positive samples using Espline SARS-CoV-2 (Fujirebio, Tokyo, Japan) according to the manufacturer’s instructions. Lumipulse SARS-CoV-2 Ag kit^®^ (Fujirebio, Tokyo, Japan), a sandwich CLEIA using SARS-CoV-2 N-Ag monoclonal antibodies on LUMIPULSE G1200 (Fujirebio, Tokyo, Japan), was performed as described [7]. Antigen levels of >0.67 pg/mL were defined as positive according to the manufacturer’s preliminary analysis (data not shown). Statistical analyses were conducted by R 4.0.2 (R Foundation for Statistical Computing, Vienna, Austria), and Clopper–Pearson exact confidence interval was used for a proportion. This study was approved by the Institutional Ethics Board (020-0116), and informed consent was obtained from all patients.

## 3. Results

SARS-CoV-2 positive samples included 17 NPS and 17 saliva samples. The median time of sampling was 9 days (range, 2–14 days) after symptom onset. PCR positivity was again confirmed after thawing in all samples. ICA detected viral antigens in only 14 (41%, 95% confidential interval [CI]: 25–59%) samples (Table 1). In particular, positive results for the virus were only 24% (95% CI: 7–50%) in the saliva samples in contrast to 59% (95% CI: 33–82%) positivity in the NPS. Of note, ICA was positive in five (83%, 95% CI: 36–100%) out of six samples collected within four days after symptom onset and in nine (32%, 95% CI: 16–52%) of twenty-eight samples collected thereafter. In NPS, tests were positive in nine (82%, 95% CI: 48–98%) out of eleven samples collected within ten days after symptom onset, but in just one (17%, 95% CI: 0–64%) of six samples collected thereafter. In saliva, all three samples collected at two–four days after symptom onset were positive, but only one (7%, 95% CI: 0–34%) of fourteen samples collected thereafter were positive.

On the other hand, in PCR-positive samples CLEIA yielded 91% (95% CI: 76–98%) positivity, with 82% (95% CI: 57–96%) positivity in saliva and 100% (95% CI: 80–100%) positivity in NPS. CLEIA yielded 99.4% (95% CI: 97.7–99.9%) negativity in PCR-negative samples. However, three out of thirty-four samples were CLEIA-negative. These samples were all saliva collected at 7, 12, and 14 days after symptom onset, with cycle threshold (Ct) values of 32.4–33.8 by PCR.

**Figure 1:**
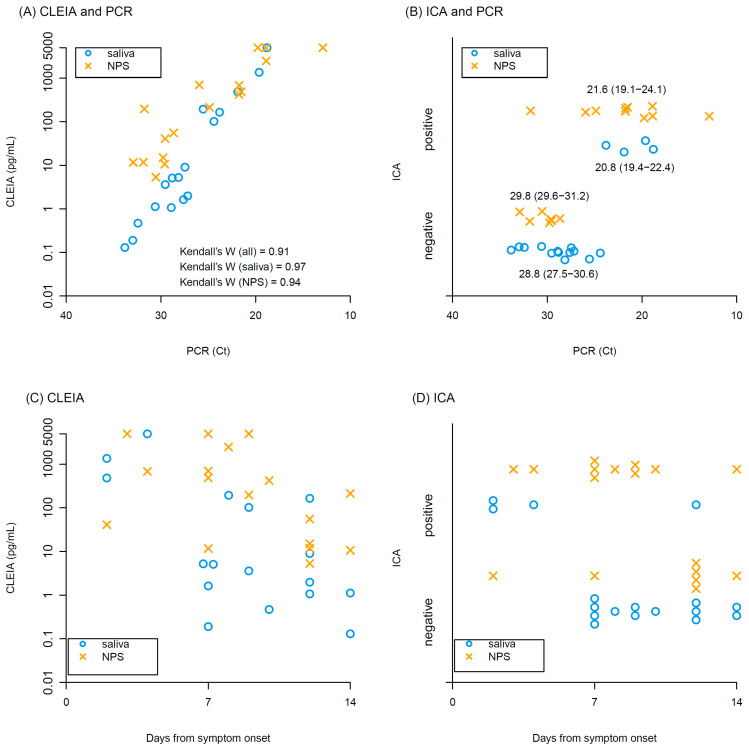
Results of CLEIA and ICA. Relationships between (**A**) antigen concentrations of CLEIA and Ct values of PCR, and (**B**) ICA positivity/negativity and Ct values of PCR. Median and interquartile range of Ct values are shown in (**B**). Relationship between days from symptom onset and (**C**) antigen concentrations of CLEIA, (**D**) Ct values of PCR. Blue circles and yellow crosses represent saliva and NPS samples, respectively.

Kendall’s coefficient of concordance of antigen concentrations with CLEIA against Ct values of PCR was 0.91, indicating high correlation between CLEIA and PCR in both saliva and NPS (Figure 1A). ICA positivity tended to have higher viral loads of PCR (Ct values: 21.6 (interquartile range, IQR): 19.1–23.3 in ICA positive vs 29.6 (IQR: 28.0–30.9) in ICA negative), but many samples were ICA negative, particularly in the saliva samples (Figure 1B). Antigen concentrations determined by CLEIA declined over time after symptom onset (Figure 1C). Similarly, the frequency of ICA positivity decreased over time (Figure 1D).

**Figure 2:**
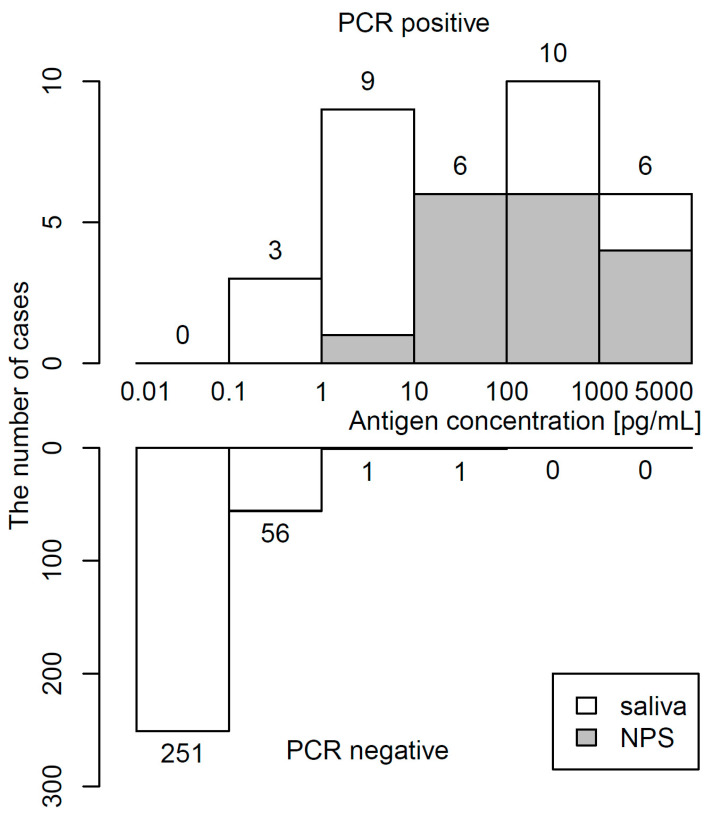
Histogram of antigen concentration. Upper and lower panels show frequency of PCR-positivity and PCR-negativity, respectively.

The distribution of antigen concentrations determined by CLEIA in 34 PCR-positive and 309 PCR-negative samples is shown in Figure 2. The median (IQR) antigen concentration was 48.2 (5.2–486.7) pg/mL in PCR-positive specimens and 0.03 (0.01–0.09) pg/mL in PCR-negative specimens. The maximum of the antigen concentration in PCR-negative specimens was 24.23 pg/mL. Raw data in PCR-positive specimens are shown in Table S1.

## 4. Discussion

Our results suggest that ICA can be used only within 4 days of symptom onset using both NPS and self-collected saliva. However, ICA is not reliable in samples collected thereafter with high false-negative rates, particularly in saliva. It is well documented that SARS-CoV-2 tends to persist longer in NPS than in saliva [8]. Thus, it is reasonable to speculate that the lower sensitivity of saliva ICA is due to late sampling rather than the difference in antigen load between saliva and NPS. It should be noted that ICA of influenza is also recommended to perform within 3 days of symptom onset [9]. However, these results should be confirmed in larger cohort studies. A major limitation of our study is that samples were frozen and thawed before testing. Our previous studies addressing the effects of freezing and thawing on viral testing showed that freeze–thaw did not significantly affect the Ct values of PCR, while it significantly reduced antigen and culture titers by about 25% [10,11].

Nonetheless, in contrast, we have shown that CLEIA using saliva is much more reliable and accurate, with high correlation observed between antigen concentrations and RNA load by PCR [7]. However, there were three PCR-positive but CLEIA-negative samples, which were all saliva collected at 7, 10, and 14 days after symptom onset, with Ct values of 32.4–33.8 by PCR. A “positive” PCR result does not necessarily indicate presence of live virus. Patients with Ct values above 33–34 by PCR were unlikely to be infectious [12,13]. We therefore recommend using saliva for CLEIA only in patients who have developed symptoms within a week. Vice versa, two (0.65%) of three hundred and nine samples were PCR-negative but CLEIA-positive with high antigen concentrations of 8.45 and 24.23 pg/mL (Table S2). Reexamination of these specimens confirmed CLEIA-positivity. This could reflect a false positive CLEIA, but the possibility of a false negative PCR result cannot be completely ruled out [14], and the clinical implication of this discrepancy remains to be elucidated. Antigen detection of SARS-CoV-2 yields results quickly. However, the use of a rapid antigen test should be limited to within a few days after symptom onset. CLEIA using self-collected saliva have already been implemented at Japanese airport quarantine to facilitate expeditious processing of international travelers [15].

## Figures and Tables

**Table 1:** Diagnostic results in positive specimens diagnosed by RT-PCR.

Test	Positive (%, 95% Confidence Interval)		
	Total (*n* = 34)	Saliva (*n* = 17)	NPS (*n* = 17)
ICA	14 (41%, 25–59%)	4 (24%, 7–50%)	10 (59%, 33–82%)
CLEIA	31 (91%, 76–98%)	14 (82%, 57–96%)	17 (100%, 80–100%)

## Data Availability

All raw data are available in Tables S1 and S2.

## Supplementary Materials

The following are available online at https://www.mdpi.com/article/10.3390/idr13030069/s1, Table S1: Raw data in PCR-positive specimens, Table S2: Antigen concentration by CLEIA in PCR-negative specimens.

## References

[1] Wyllie A.L., Fournier J., Casanovas-Massana A., Campbell M., Tokuyama M., Vijayakumar P., Warren J.L., Geng B., Muenker M.C., Moore A.J. (2020). Saliva or nasopharyngeal swab specimens for detection of SARS-CoV-2. N. Engl. J. Med..

[2] Yokota I., Shane P.Y., Okada K., Unoki Y., Yang Y., Inao T., Sakamaki K., Iwasaki S., Hayasaka K., Sugita J. (2021). Mass screening of asymptomatic persons for SARS-CoV-2 using saliva. Clin. Infect. Dis..

[3] Bastos M.L., Perlman-Arrow S., Menzies D., Campbell J.R. (2021). The sensitivity and costs of testing for SARS-CoV-2 infection with saliva versus nasopharyngeal swabs: A systematic review and meta-analysis. Ann. Intern. Med..

[4] Yokota I., Hattori T., Shane P.Y., Konno S., Nagasaka A., Takeyabu K., Fujisawa S., Nishida M., Teshima T. (2021). Equivalent SARS-CoV-2 viral loads by PCR between nasopharyngeal swab and saliva in symptomatic patients. Sci. Rep..

[5] Nagura-Ikeda M., Imai K., Tabata S., Miyoshi K., Murahara N., Mizuno T., Horiuchi M., Kato K., Imoto Y., Iwata M. (2020). Clinical evaluation of self-collected saliva by RT-qPCR, direct RT-qPCR, RT-LAMP, and a rapid antigen test to diagnose COVID-19. J. Clin. Microbiol..

[6] Hirotsu Y., Maejima M., Shibusawa M., Nagakubo Y., Hosaka K., Amemiya K., Sueki H., Hayakawa M., Mochizuki H., Tsutsui T. (2020). Comparison of automated SARS-CoV-2 antigen test for COVID-19 infection with quantitative RT-PCR using 313 nasopharyngeal swabs, including from seven serially followed patients. Int. J. Infect. Dis..

[7] Yokota I., Shane P.Y., Okada K., Unoki Y., Yang Y., Iwasaki S., Fujisawa S., Nishida M., Teshima T. (2021). A novel strategy for SARS-CoV-2 mass screening with quantitative antigen testing of saliva: A diagnostic accuracy study. Lancet Microbe.

[8] Iwasaki S., Fujisawa S., Nakakubo S., Kamada K., Yamashita Y., Fukumoto T., Sato K., Oguri S., Taki K., Senjo H. (2020). Comparison of SARS-CoV-2 detection in nasopharyngeal swab and saliva. J. Infect..

[9] Green D.A., StGeorge K. (2018). Rapid antigen tests for influenza: Rationale and significance of the FDA reclassification. J. Clin. Microbiol..

[10] Fukumoto T., Iwasaki S., Fujisawa S., Hayasaka K., Sato K., Oguri S., Taki K., Nakakubo S., Kamada K., Yamashita Y. (2020). Efficacy of a novel SARS-CoV-2 detection kit without RNA extraction and purification. Int. J. Infect. Dis..

[11] Oguri S., Fujisawa S., Kamada K., Nakakubo S., Yamashita Y., Nakamura J., Horii H., Sato K., Nishida M., Teshima T. (2021). Effect of varying storage conditions on diagnostic test outcomes of SARS-CoV-2. J. Infect..

[12] Bullard J., Dust K., Funk D., Strong J.E., Alexander D., Garnett L., Boodman C., Bello A., Hedley A., Schiffman Z. (2020). Predicting infectious severe acute respiratory syndrome coronavirus 2 from diagnostic samples. Clin. Infect. Dis..

[13] Wolfel R., Corman V.M., Guggemos W., Seilmaier M., Zange S., Muller M.A., Niemeyer D., Jones T.C., Vollmar P., Rothe C. (2020). Virological assessment of hospitalized patients with COVID-2019. Nature.

[14] Kucirka L.M., Lauer S.A., Laeyendecker O., Boon D., Lessler J. (2020). Variation in false-negative rate of reverse transcriptase polymerase chain reaction-based SARS-CoV-2 tests by time since exposure. Ann. Intern. Med..

[15] Yokota I., Shane P.Y., Teshima T. (2021). Logistic advantage of two-step screening strategy for SARS-CoV-2 at airport quarantine. Travel Med. Infect. Dis..

