# Supplementary material for: Performance of Qualitative and Quantitative Antigen Tests for SARS-CoV-2 Using Saliva
Source: Infect Dis Rep. 2021 Aug 24;13(3):742–7. doi: 10.3390/idr13030069 (PMC8395849; doi:10.3390/idr13030069)
Supplement: Supplementary file 1 [file idr-13-00069-s001.zip › idr-1337346-supplementary.pdf]

Table S1. Raw data in PCR-positive specimens

Days after symptom onset	specimen	Ct value	Tp value (sec)	CLEIA (pg/mL)	ICA
2	NPS	29.56192	586	40.87	negative
3	NPS	12.90119	301	5000	positive
4	NPS	21.74256	391	685.64	positive
7	NPS	18.89330	346	5000	positive
7	NPS	21.52387	391	487.16	positive
7	NPS	25.95481	451	699.01	positive
7	NPS	32.91834	661	11.62	negative
8	NPS	18.88139	436	2484.37	positive
9	NPS	19.76842	346	5000	positive
9	NPS	31.75552	646	196.65	positive
10	NPS	21.75409	391	422.62	positive
12	NPS	31.85005	526	11.77	negative
12	NPS	30.54592	601	5.34	negative
12	NPS	29.77066	511	15.02	negative
12	NPS	28.65936	556	55.43	negative
14	NPS	24.86924	406	213.09	positive
14	NPS	29.59635	556	10.62	negative
2	saliva	19.62641	391	1357.72	positive
2	saliva	21.89011	376	485.26	positive
4	saliva	18.79502	511	5000	positive
7	saliva	28.14606	526	5.26	negative
7	saliva	32.96928	751	0.19	negative
7	saliva	27.62087	541	1.63	negative
7	saliva	28.81459	571	5.12	negative
8	saliva	25.55043	436	194.68	negative
9	saliva	24.40270	481	102.21	negative
9	saliva	29.54057	526	3.62	negative
10	saliva	32.43248	496	0.47	negative
12	saliva	28.90566	571	1.07	negative
12	saliva	27.46050	586	9.06	negative
12	saliva	27.17160	616	1.98	negative
12	saliva	23.80877	496	164.85	positive
14	saliva	33.81876	601	0.13	negative
14	saliva	30.60193	601	1.12	negative

Table S2. Antigen concentration by CLEIA in PCR-negative specimens

Antigen concentration (pg/mL)	Frequency	Antigen concentration (pg/mL)	Frequency
0.01	122	0.19	1
0.02	18	0.20	2
0.03	19	0.21	2
0.04	16	0.22	1
0.05	14	0.23	2
0.06	12	0.24	1
0.07	6	0.25	1
0.08	19	0.27	1
0.09	11	0.28	1
0.10	14	0.30	1
0.11	5	0.34	2
0.12	3	0.37	1
0.13	6	0.39	1
0.14	7	0.40	2
0.15	3	0.60	1
0.16	6	0.63	1
0.17	4	8.45 (4.78)*	1
0.18	1	24.23 (16.07)*	1

* The antigen concentration at re-test was shown in parenthesis.
